# Modulation of Photocatalytic CO_2_ Reduction by *n*–*p* Codoping Engineering of Single-Atom Catalysts

**DOI:** 10.3390/nano14141183

**Published:** 2024-07-11

**Authors:** Guowei Yin, Chunxiao Zhang, Yundan Liu, Yuping Sun, Xiang Qi

**Affiliations:** 1 School of Physics and Optoelectronic Engineering, Shandong University of Technology, Zibo 255000, China; 2 Hunan Key Laboratory of Micro-Nano Energy Materials and Devices, Xiangtan University, Xiangtan 411105, China

**Keywords:** *n*–*p* codoping, single-atom catalysts, Coulomb interactions, *d*-band center, first-principles calculation

## Abstract

Transition metal (TM) single-atom catalysts (SACs) have been widely applied in photocatalytic CO_2_ reduction. In this work, *n*–*p* codoping engineering is introduced to account for the modulation of photocatalytic CO_2_ reduction on a two-dimensional (2D) bismuth-oxyhalide-based cathode by using first-principles calculation. *n*–*p* codoping is established via the Coulomb interactions between the negatively charged TM SACs and the positively charged *Cl* vacancy (*V_Cl_*) in the dopant–defect pairs. Based on the formation energy of charged defects, neutral dopant–defect pairs for the Fe, Co, and Ni SACs (*P_TM_*^0^) and the −1*e* charge state of the Cu SAC-based pair (*P_Cu_*^−1^) are stable. The electrostatic attraction of the *n*–*p* codoping strengthens the stability and solubility of TM SACs by neutralizing the oppositely charged *V_Cl_* defect and TM dopant. The *n*–*p* codoping stabilizes the electron accumulation around the TM SACs. Accumulated electrons modify the *d*-orbital alignment and shift the *d*-band center toward the Fermi level, enhancing the reducing capacity of TM SACs based on the d-band theory. Besides the electrostatic attraction of the *n*–*p* codoping, the *P_Cu_*^−1^ also accumulates additional electrons surrounding Cu SACs and forms a half-occupied *d_x_*^2^_−*y*_^2^ state, which further upshifts the *d*-band center and improves photocatalytic CO_2_ reduction. The metastability of *Cl* multivacancies limits the concentration of the *n*–*p* pairs with *Cl* multivacancies (*P_TM@nCl_* (n > 1)). Positively charged centers around the *P_TM@nCl_* (n > 1) hinders the CO_2_ reduction by shielding the charge transfer to the CO_2_ molecule.

## 1. Introduction

Artificial photocatalytic CO_2_ reduction is an intriguing research area aiming to reduce fossil consumption and mitigate the greenhouse effect [1,2]. Photocatalysts are the crucial materials for readily converting CO_2_ into environmentally friendly fuels via photolysis using abundant solar energy [3,4,5]. With maximal atom-utilization efficiency, transition metal (TM) single-atom photocatalysts (SACs) exhibit excellent catalytic performance comparable to precious metals [6,7,8,9,10,11]. Due to its sufficient optical absorption and adequate activation centers, two-dimensional (2D) bismuth oxyhalide BiOX (X = F, Cl, Br, I) is an outstanding host to anchor the TM SACs photocatalyst and accelerate the CO_2_ reduction [12,13,14,15,16]. For TM-doped bismuth oxyhalide, the dopant of TM SACs can form impurity levels in the forbidden band, promoting the generation and separation of photogenerated carriers [17,18]. However, the most outstanding advantage is that doped TM SACs regulate the surface state, which enhances the CO_2_ adsorption by promoting the charge transfer between the activation centers and absorbed CO_2_ molecules [17,18,19]. For example, isolated Cu SACs on BiOBr (Cu@BiOBr) establish a strong built-in electric field which serves as an electron trap to facilitate charge transfer and stabilize charge carriers. As a result, 0.5% Cu@BiOBr has a higher CO_2_ absorption uptake (2.7 cm^3^ g^−1^) than BiOBr (2.3 cm^3^ g^−1^) [20]. In Co-SAC-doped Bi_3_O_4_Br, the isolation of Co^2+^ by replacing Bi^3+^ enables Co@Bi_3_O_4_Br layers to be more negatively charged, improving the CO_2_ adsorption and stabilizing the *COOH intermediate on the surface [17]. Charge localization induced by Fe SAC doping strengthens CO_2_ bonding strengths and improves the CO_2_ absorptive capacity in both porous Bi_5_O_7_I Micro-flower and B_i4_O_5_I_2_ [21,22].

Some issues remain to be addressed in understanding the CO_2_ photolysis by the TM SACs on BiOX. The defect configurations around the TM SACs are ambiguous, especially whether halogen atoms are present or not near the TM impurity sites [17,18,19,20,21]. Both experimental and theoretical works confirm that the [Bi_2_O_2_]^2+^ layer is sandwiched by two X^−^ layers in BiOX [23,24,25,26,27,28,29]. However, the unlocked bismuth (Bi) surface without a halogen covering is usually used to simulate photocatalytic CO_2_ reduction around the TM SACs [17,18,19,20]. This divergence highlights the necessity of assessing the effects of halogen vacancy (*V_halogen_*) on the microstructures and reduction performance around the TM SACs. *V_halogen_* usually acts as an *n*-type defect, whereas TM SACs exhibit *p*-type characteristics and form negatively charged centers to promote the reduction reaction [23,24].

The *n*–*p* codoping concept is established based on the electrostatic attraction between the *n-* and *p*-type dopants (or defects) with opposite charge states, which affects the ionization, solubility, and charge transfer of the doped semiconductors [30,31,32,33]. For example, the electrostatic attraction within the *n*–*p* pair enhances both thermodynamic and kinetic solubilities, creating tunable intermediate bands to effectively narrow the band gap and enhance the visible-light photoactivity of TiO_2_ [32]. The *n*–*p* pairs limit the applications of Ga_2_O_3_ by affecting the dopant ionization [33]. On BiOX, *n*–*p* codoping can be established via the electrostatic interactions in the combination of *p*-type TM dopants and *n*-type *V_halogen_* defects. The effects of *n*–*p* codoping on the stability and reducing capacity of TM SACs must be determined. For TM SACs, the *d*-band theory is universally recognized as a means of evaluating the reducing capacity [34,35]. Consequently, the dependence of the orbital alignment and *d*-band center of TM_*d* on *n*–*p* codoping deserves systematic study regarding the CO_2_ reduction on TM SACs on BiOX.

In this paper, we investigate the effects of *n*–*p* codoping on the stability and reducing capacity of TM SACs for photocatalytic CO_2_ reduction reaction (CO_2_RR) on BiOCl by using first-principles calculation. Previous experimental works found that Fe, Co, Ni, and Cu are all effective SACs for accelerating CO_2_RR on BiOX [17,18,19,20]. Consequently, we constructed a dopant–defect combination by pairing these TM SACs and their surrounding *Cl* vacancies. Thermodynamically, the balance between *n*- and *p*-defects is determined by the defect equilibrium, which can be solved by calculating the formation energies of charged defects (Δ*H_f_*) [36,37,38]. Based on the Δ*H_f_*, we find that the *Cl* monovacancy (*V_Cl_*) is more stable than the *Cl* multivacancies. *n*–*p* codoping is established by the coulomb attraction between the negatively charged *p*-type TM SACs and positively charged *n*-type *V_Cl_*. The electrostatic attraction of *n*–*p* codoping enhances the stability of TM SACs. Neutral pairs are formed for the Fe, Co, and Ni SACs (*P_TM_*^0^), while the Cu SAC-based pair is dominated by the q = −1 charge state (*P_Cu_*^−1^). In the *n*–*p* pairs, electrostatic interaction settles the electron accumulation around TM SACs. Accumulated electron occupies localized TM_*d* orbital and upshifts the *d*-band center toward the Fermi level, enhancing the reducing capacity of TM SACs based on the d-band theory. As a result, the CO_2_ absorption is improved along with the enhancement of charge transfer and the decrease in Gibbs free energies. The *P_Cu_*^−1^ also locates additional electrons surrounding the Cu SACs and forms a half-occupied *d_x_*^2^*_−y_*^2^ state, further unshifting the *d*-band center and improving the reducing capacity of TM SACs. The metastability of *Cl* multivacancies limits the concentration of the *n*–*p* pairs with *Cl* multivacancies (*P_TM@nCl_* (n > 1)). Positively charged centers around *P_TM@nCl_* (n > 1) shield the charge transfer between the *P_TM@nCl_* (n > 1) and the CO_2_ molecule, hindering the CO_2_ reduction.

## 2. Computational Details

Density functional theory (DFT) [39] calculations were performed in the Vienna Ab initio Simulation Package (VASP) [40]. The interaction of ions with electrons was determined by projector augmented wave potentials (PAWs) [41]. The electron exchange–correlation energy was treated based on local-density approximation (LDA) [42] because the lattice constants and band structure of BiOCl calculated by LDA are in good agreement with experimental values [43]. The results were also checked based on the Perdew–Burke–Ernzerhof (PBE) functional [44]. The kinetic energy cutoff energy was 500 eV, and the total energy convergence criterion was set to 10^−5^ eV. A 4 × 4 × 1 supercell with over 15 Å of lattice constant was chosen to avoid the interactions of adjacent point defects. The K-mesh was optimized based on the minimum energy principle, and 3 × 3 × 1 Monkhorst–Pack grids were found to be sufficient for sampling the Brillouin zone for the supercell. A vacuum space of over 15 Å was applied to avoid the interactions of neighbor images along the z direction.

To solve the defect equilibrium between TM dopants and the compensating *V_Cl_*, the formation energy of charged defects was calculated with the following formula [45,46]:

(1) $$\Delta H_{f}=E_{tot}(q,\alpha )-E_{tot}(host)+\sum_{i}n_{i}(E_{i}+\mu_{i})+q(\varepsilon_{VBM}+E_{f})$$

where *E_tot_*(*q*,*α*) and *E_tot_*(*host*) are the total energy of the defect system and pristine BiOCl, respectively; *n* is the number of Cr, Bi, and TM dopants; *q* is the number of electrons transferred from supercell to reservoirs in forming the defect; and *μ_i_* is the chemical formula of constituent *i* with energy *E_i_*. *E_f_* is the Fermi energy with respect to the valence band maximum (VBM) and ranges from the VBM to the conduction band minimum (CBM). The chemical potentials of Bi (*μ_Bi_*) and TM (*μ_TM_*) were derived from the energies of their corresponding metals. The chemical potentials of O and Cl were limited to avoid the formation of elementary substances and maintain a stable BiOCl compound. *μ_Cl_* and *μ*_O_ are defined as:

(2) $$\Delta H_{f}(BiOCl)=2\mu_{Bi}+2\mu_{Cl}+2\mu_{O},$$

(3) $$\mu_{Cl}\leq 0,$$

where Δ*H*_BiOCl_ is the formation energy of BiOCl. Under *Cl*-poor conditions, the chemical potential of the *Cl* atom is defined as the energy in the species *Cl*^+^ (−5.36 eV). The total energies of the charged systems should be corrected for the interaction of the charged defect with the compensating background and its periodic images. We used Makov–Payne (M-P) corrections, formulated as q2 α/2Ɛ L, where L is the linear dimension of the supercell, Ɛ is the static dielectric constant, and α is the Madelung constant.

To evaluate the reaction coordinate of CO_2_ reduction, Nørskov’s method [47] was used to calculate the Gibbs free energy difference:

(4) $$\Delta G=\Delta E+\Delta E_{ZPE}+T\Delta S$$

∆*E*, ∆*E_ZPE_*, and ∆*S* denote the absorbed energy difference, the zero-point energy difference, and the entropy difference between the adsorbed state and the corresponding free state for density functional theory calculations, respectively. *T* is the temperature of the system, 298.15 K.

The reactions of CO_2_ adsorption and the *CO_2_ plus H formation *COOH process are defined as follows [48]:

(5) $$*+CO_{2}\to *CO_{2}$$

(6) $$*CO_{2}+H^{+}+e^{-}\to *COOH$$

where * represents adsorption sites, and *CO_2_ and *COOH represent adsorption intermediate states. Therefore, the CO_2_RR is calculated as:

(7) $$\Delta G_{CO_{2}*}=G_{CO_{2}*}-G_{*}-G_{CO_{2}}$$

(8) $$\Delta G_{COOH*}=G_{COOH*}-\frac{1}{2}G_{H_{2}}-G_{CO_{2}*}$$

Charge density redistribution is determined by the charge density difference between the pairs and corresponding isolated systems [49]. The charge density difference is calculated as follows:

(9) $$\Delta \rho =\rho_{AB}-\rho_{A}-\rho_{B}$$

where *ρ_A_* and *ρ_B_* are the charge densities of isolated systems A and B, respectively, and *ρ_AB_* is the charge density of the pairs.

## 3. Results and Discussion

### 3.1. Structures and Stability

**Figure 1:**
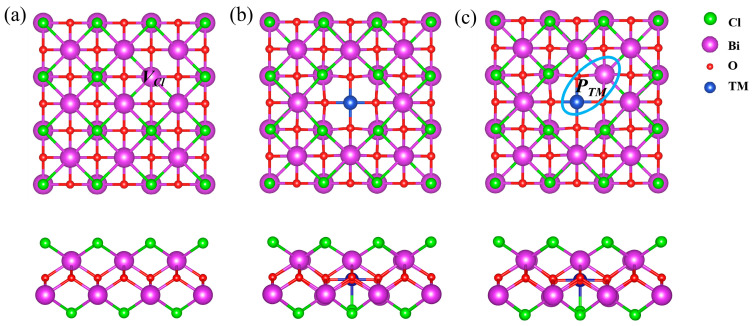
Schematic diagrams of atomic structures for 2D BiOCl with (**a**) *V_Cl_*, (**b**) *Bi_TM_*, and (**c**) *P_TM_*. Blue circle refer to the *P_TM_*.

In stable BiOCl, the [Bi_2_O_2_]^2+^ layer is sandwiched by two Cl^−^ layers (Figure 1), and the lattice constant is calculated to be 3.854 Å, agreeing with previous works [13,50,51]. The TM SACs are doped on the BiOX by substituting a bismuth atom based on experiments [16,17,18,19,20]. The TM SAC dopants are denoted as the *Bi_TM_* on the intrinsic BiOX surface as shown in Figure 1b. The dopant–defect pair (*P_TM@_*_n*Cl*_) comprises the *Bi_TM_* and its surrounding Cl vacancy as shown in Figure 1. The *P_TM@3Cl_* is the combination of a TM SAC and Cl trivacancy. In the Cl trivacancy, the proportion of unlocked Bi atoms is up to 19%, which is large enough to comprise the unlocked Bi region. The structures of the *Bi_TM_* and *P_TM@_*_1*Cl*_ (*P_TM_* for short) are given in Figure 1b,c, and the *P_TM@nCl_* (*n* > 1) is shown in Figure S1. To assess the stability of the pairs, the formation energy (Δ*H_f_*) of charged defects is investigated as a function of *E_f_* based on Equation (1). The cathode where the CO_2_RR takes place is an electron reservoir and corresponds to the *n*-type semiconductor. Therefore, we focus on the electron-rich (*e*-rich) condition where the *E_f_* is close to the CBM. The Δ*H_f_* calculated following Equation (1) also depends on *μ_Cl_*. We focus on the *Cl*-rich limit (*μ_Cl_* = 0 eV), considering that the electrolyte solution usually accelerates the surface Cl ion exchange in photocatalytic experiments.

Based on previous experiments, surface halogen atoms significantly influence the charge transfer between activation sites and absorbed molecules [52,53]. The stability of Cl vacancies is evaluated prior to the dopant–defect pairs. Figure S2a illustrates the formation energy of the Cl monovacancy *V_Cl_*, Cl di-vacancy (*V*_2*Cl*_), and tri-vacancy *V_Cl_* (*V*_3*Cl*_) with varied charge states as a function of *E_f_*. The *E_f_* ranges from the VBM and CBM. One can see that the *V_Cl_* is energy favorable in these three kinds of Cl vacancies if *E_f_* is above the mid-gap (i.e., for the *e*-rich condition). In the *V*_3*Cl*_, 3/16 of Bi atoms are uncovered by the surface Cl atoms so that the *V*_3*Cl*_ is large enough with regard to the unlocked Bi region. Consequently, the *V_Cl_* is more stable than the unlocked Bi region in the BiOCl cathode. The *V_Cl_* is capable of forming a donor defect with *q =* +1 charge state (*V_Cl_*^+1^, with the superscript referring to the charge state) when the *E_f_* is in most of the gap and becomes a neutral defect only when the *E_f_* is closer to the CBM. The charge transition level Ɛ(+1/−1) is only 0.07 eV above the CBM, indicating that it is a donor defect. The Fermi level of the *V_Cl_*^0^ is located in the conduction band in the spin–polarized density of states, which also indicates the donor defect characteristic.

**Figure 2:**
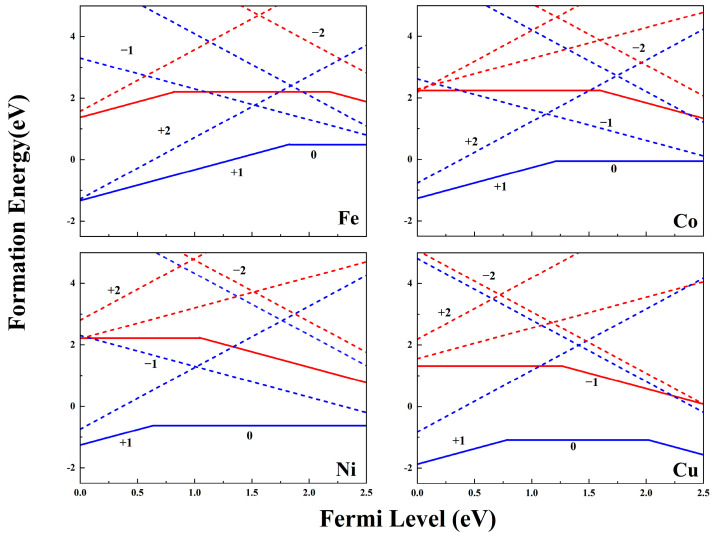
Formation energies of *Bi_TM_* (red lines) and *P_TM_* (blue lines) at the *Cl*-rich limit for Fe, Co, Ni, and Cu. The numerical notations refer to charge states. The dotted lines refer to the formation energies of meta-stable charged defects.

Considering the stability of the *V_Cl_*, we first investigate the *P_TM_*, which pairs the *Bi_TM_* and surrounding single *V_Cl_*, as shown in Figure 1c. The formation energy Δ*H_f_* is calculated to investigate the stability and ionization of the *P_TM_* and *Bi_TM_*. If *E_f_* is close to the CBM, as shown in Figure 2, the *Bi_TM_*^−1^ dominates the *Bi_TM_* for all the Fe, Co, Ni, and Cu SACs, indicating the *p*-type impurities characteristic of these TM SACs on pristine BiOCl in the *e*-rich condition. When the positively charged *V_Cl_*^+1^ and negatively charged *Bi_TM_*^−1^ move close to each other, as shown in Figure 1c, *n*–*p* codoping is established due to the coulomb attraction and forms the *P_TM_* pairs. Due to the electrostatic attraction of the *n*–*p* codoping, the Δ*H_f_* of the *P_TM_* is lower than the relative *Bi_TM_* as shown in Figure 2. Consequently, the *V_Cl_* facilitates the stability of the TM SACs on the BiOCl. When *E_f_* is close to the CBM, neutral pairs (*P_TM_*^0^) are energetically favorable for the Fe, Co, and Ni SACs, while the negatively charged *P_Cu_*^−1^ with charge state *q* = −1 is most stable for the Cu SACs. The *P_Cu_*^−1^ possesses the lowest *E_f_* (as low as −1.56 eV) in all the defects, suggesting that the *P_Cu_*^−1^ can exist in great quantities on the BiOX cathode. The *q* = −1 charge state indicates that the *P_Cu_*^−1^ further traps electrons from the surrounding lattice besides the inner electrostatic interaction in the pairs. The *Cl*-poor condition, where Δ*H_f_* is elevated by 5.36 eV depending on the *μ_Cl_*, as shown in Figure S3, hinders the TM SACs from anchoring on the BiOCl.

**Figure 3:**
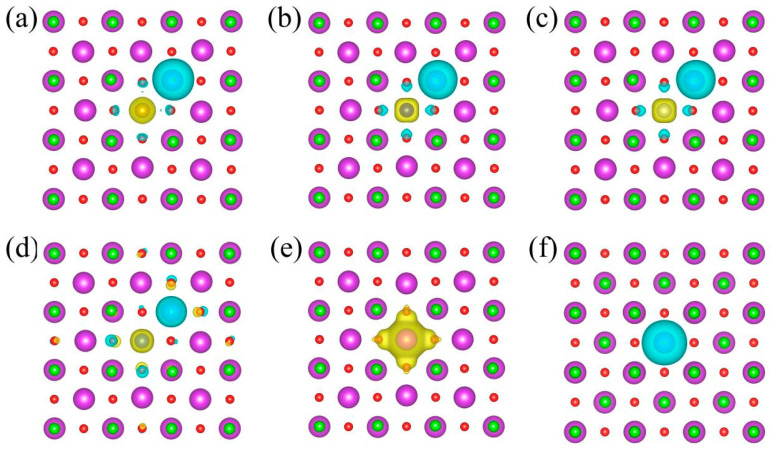
Charge density redistribution of (**a**) *P_Fe_*^0^, (**b**) *P_Cu_*^0^, (**c**) *P_Ni_*^0^, and (**d**) *P_Cu_*^0^. (**e**,**f**) The charge density distribution of the isolated *Bi_Cu_*^−1^ and *V_Cl_*^−1^. The yellow (blue) isosurface refers to the charge accumulation (dissipation) region. The isovalue is set to 0.03 *e*/Å.

The electrostatic interaction is the key factor for establishing *n*–*p* codoping. We investigate the electrostatic interaction in the pairs by calculating the charge density redistribution. The charge density redistribution is determined as the charge density difference of the BiOCl system after and before forming *P_TM_* pairs. In Figure 3, the yellow and blue isosurfaces refer to the electron accumulation and dissipation regions, respectively. Before combining into *P_TM_* pairs, isolated TM SACs are all negatively charged, and *V_Cl_* is positively charged based on the Δ*H_f_* in Figure 2 and Figure S3a. The negative charge density localizes around the TM SACs in the *Bi_TM_*, while the positive charge localizes around the vacancy site in the *V_Cl_* as shown in Figure 3e,f. In all the *P_TM_* pairs, negative and positive charges also separated at the adjacent TM SACs and *V_Cl_* site as shown from Figure 3a–d. An obvious coulomb interaction is elicited between the closer positive and negative charges, forming more stable *n*–*p* codoping.

### 3.2. Modulation of CO_2_ Reduction

**Figure 4:**
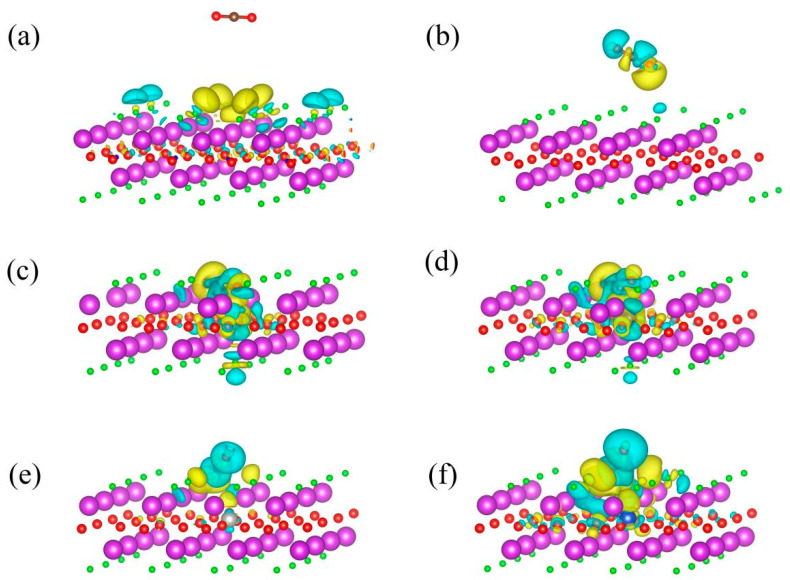
Charge transfer between absorbed CO_2_ and the (**a**) pristine BiOCl, (**b**) *V_Cl_*^+1^, (**c**) *P_Fe_*^0^, (**d**) *P_Co_*^0^, (**e**) *PNi*0, and (**f**) *P_Cu_*^−^^1^. The isovalue is set to 0.0003 *e*/Å.

**Figure 5:**
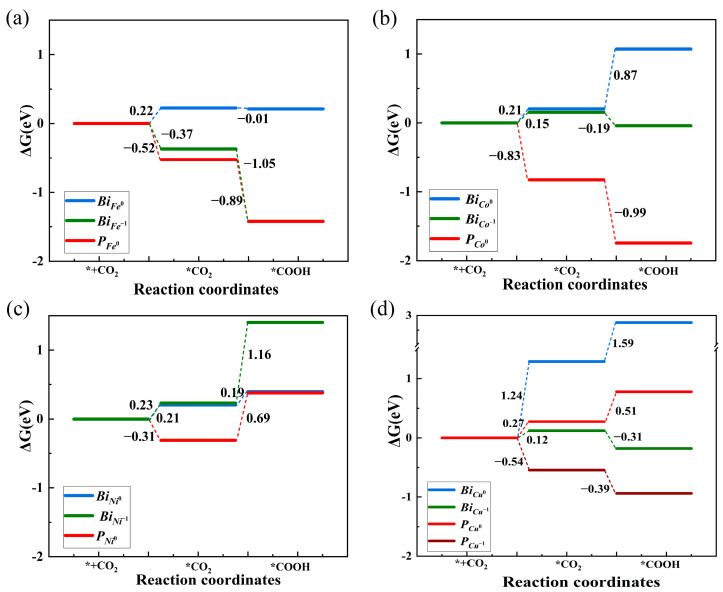
Gibbs free energy (ΔG) of CO_2_ absorption and *CO_2_ hydrogenation (*COOH) at (**a**) Fe, (**b**) Co, (**c**) Ni, and (**d**) Cu SAC sites.

CO_2_ absorption (*CO_2_) is usually the rate-determining step in the CO_2_RR [15,18]. We investigate CO_2_ absorption on defective BiOCl systems to evaluate the effects of *n*–*p* codoping on the photocatalytic CO_2_RR. From Figure 4a, one can see that the CO_2_ is physically absorbed on the pristine BiOCl surface, and hardly any charges transfer between the BiOCl and the CO_2_. Only the surface charge density distribution of the BiOCl is disturbed by absorbed CO_2_. The charge transfer between the *V_Cl_*^+1^ site and absorbed CO_2_ is enhanced, but still only physical absorption occurs with over 3.5 Å of distance as shown in Figure 4b. The Gibbs free energy difference (ΔG) is also calculated based on Equations (5) and (6) to evaluate the CO_2_ absorption. Negative ΔG refers to the chemisorption of CO_2_ on the photocatalyst. In Figure S4, one can see that the ΔG is 0.16 eV on the *V_Cl_*^+1^, lower than that on pristine BiOCl (0.32 eV). Consequently, the *V_Cl_*^+1^ mildly improves the CO_2_ absorption. For the CO_2_ absorption on the dopant–defect pairs, dramatic charge transfers are found in the neutral *P_Fe_*^0^, *P_Co_*^0^, and the negatively charged *P_Cu_*^−1^ as shown in Figure 4c, Figure 4d, and Figure 4e, respectively. The lengths of C–TM bonds between the absorbed CO_2_ and these pairs are 1.90 Å, 1.97 Å, and 1.97 Å as shown in Table 1, while the ΔG sharply declines to −0.52 eV, −0.83 eV, and −0.54 eV for the *P_Fe_*^0^, *P_Co_*^0^, and *P_Cu_*^−1^, respectively, as shown in Figure 5. As a result, the *n*–*p* codoping facilitates the absorption of the CO_2_ on the *P_Fe_*^0^, *P_Co_*^0^, and *P_Cu_*^−1^. The charge transfers in the *P_Ni_*^0^ are slightly weaker, and the distance between the absorbed CO_2_ and Ni SACs (3.31 Å) is larger than that in other *P_TM_*s, but the ΔG still declines to −0.31 eV. Consequently, the CO_2_ absorption is also accelerated by the *n*–*p* codoping in the *P_Ni_*^0^.

For comparison, we also evaluated the CO_2_ absorption on the isolated *Bi_TM_*^0^ and *Bi_TM_*^−1^. On the neutral *Bi_TM_*^0^, the CO_2_ is only absorbed physically on the TM SAC sites with fewer charge transfers as seen in Figure S5. The distances between the CO_2_ and *Bi_TM_*^0^ sites are all beyond 4 Å and are obviously larger than the distances between the CO_2_ and the dopant–defect pairs as shown in Table 1. The ΔGs of CO_2_ absorption on the *Bi_TM_*^0^ are all positive as shown in Figure 5. The CO_2_ is therefore still physically absorbed at the neutral *Bi_TM_*^0^ site. For the CO_2_ absorption on the negative *Bi_TM_*^−1^, we find that the CO_2_ is only chemically adsorbed at the *Bi_Fe_*^−1^ but is still physically absorbed at the *Bi_Co_*^−1^, *Bi_Ni_*^−1^, and *Bi_Cu_*^−1^. In detail, an intense charge transfer is formed between the CO_2_ and *Bi_Fe_*^−1^ site as shown in Figure S6a, while the length of the C-Fe bond is 1.90 Å as shown in Table 1. The ΔG of CO_2_ absorption is −0.18 eV in the *Bi_Fe_*^−1^. On the other hand, relatively few charge transfers are formed between the CO_2_ and *Bi_Co_*^−1^, *Bi_Ni_*^−1^, and *Bi_Cu_*^−1^ sites as shown in Figure S6b, 6c, and 6d, respectively. The distances between CO_2_ and these activation sites are over 4.0 Å as shown in Table 1. The relative ΔGs of CO_2_ absorption are 0.15 eV, 0.23 eV, and 0.12 eV in the *Bi_Co_*^−1^, *Bi_Ni_*^−1^, and *Bi_Cu_*^−1^ systems. Based on previous experiments, the carboxylate pathway is the most common route for the CO_2_RR on Fe-, CO-, and Cu-doped bismuth oxyhalide [16,17,18,19,20]. *CO_2_ hydrogenation (*COOH) follows close behind CO_2_ absorption (*CO_2_) in the carboxylate pathway and is reported to be also an important step influencing CO_2_ reduction [15,18]. We therefore also assess the ΔG of both *CO_2_ and *COOH in all defective BiOCl systems. In Figure 5, one can see that *n*–*p* codoping facilitates both the CO_2_ absorption and *CO_2_ hydrogenation with negative ΔG in the BiOCl systems by Fe, Co, and Cu SAC doping. Such an enhancement of CO_2_ photoreductions corresponds with previous experiments [18,19,20]. For the *P_Ni_*^0^-doped BiOCl, the CO_2_ absorption is exothermic, but the *CO_2_ hydrogenation is endothermic, which is related to the fact that the CO_2_ activation prefers the carbide pathway, in which the *COOH is difficult to generate [17].

### 3.3. Orbital Alignments and d-Band Center of the TM_3d

The TM_3*d* orbital is usually crucial to the reducing capacity of TM SACs [54,55]. To account for the improvement in the CO_2_ absorption by the *n*–*p* codoping, we investigate the *d*-band center of the TM_3*d* orbital states on the BiOCl systems. Based on the *d*-band theory, the *d*-band center close to the Fermi level usually facilitates the reducing capacity of TM SACs [30,31]. For comparison, the *d*-band centers of the *Bi_TM_*^0^ and *Bi_TM_*^−1^ are also calculated as shown in Table 1. One can see that the *d*-band centers of all *Bi_TM_*^0^ dopants are located far away from the Fermi level. For instance, the *d*-band centers of the *Bi_Fe_*^0^, *Bi_Ni_*^0^, and *Bi_Cu_*^0^ are as low as −2.54 eV, −3.85 eV, and −2.81 eV, respectively. Low *d*-band centers correspond to the low reducing capacity of the *Bi_TM_*^0^ and result in physical absorption of the CO_2_. For the *Bi_TM_*^−1^, the *d*-band center of *Bi_Fe_*^−1^ is significantly shifted from −2.54 eV to −1.26 eV compared with *Bi_Fe_*^0^, resulting in the enhancement from physical absorption to chemical absorption. The *d*-band center in the *Bi_Co_*^−1^ decreases from −1.81 eV to −2.45 eV, so the CO_2_ remains physically absorbed on the *Bi_Co_*^−1^ site. Although the *d*-band centers of the *Bi_Ni_*^−1^ and *Bi_Cu_*^−1^ are shifted toward the Fermi level compared with the *Bi_Ni_*^0^ and *Bi_Cu_*^0^, they are still lower than the Fermi level by −2.09 eV and −2.31 eV. Consequently, the CO_2_ is also physically absorbed on these systems. As shown in Table 1, the *d*-band centers are upshifted to −1.27, −0.83, −1.46, and −1.45 eV for the *P_Fe_*^0^, *P_Co_*^0^, *P_Ni_*^0^, and *P_Cu_*^−1^, respectively, and are comparable to those (−1.56 eV) of the Pt SACs anchored at the edge of graphene, which are excellent photocatalysts for H_2_ reduction [56]. Consequently, the modulation of CO_2_ absorption on the dopant–defect pairs derives from the enhancement of the reducing capacity of TM SACs with the upshift in the *d*-band center by the *n*–*p* codoping.

**Figure 6:**
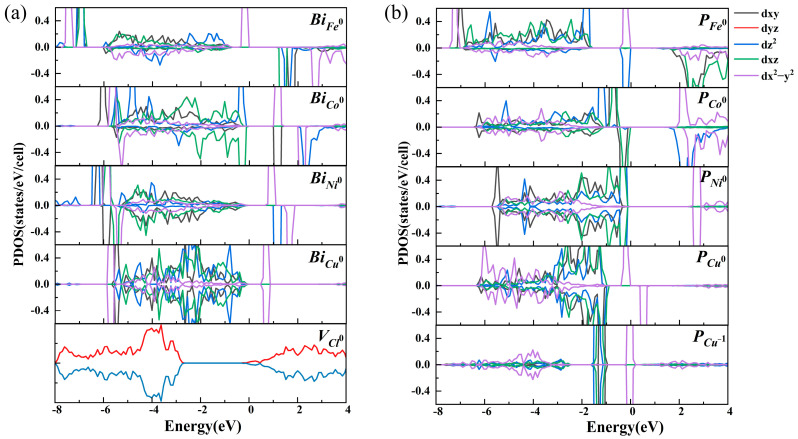
(**a**) Spin-resolved PDOS of *Bi_TM_*^0^. The spin-resolved PDOS of the *V_Cl_*^0^ is given for comparison. (**b**) Spin-resolved PDOS of the *P_TM_*^0^ and *P_Cu_*^−1^. The oxidization states are Fe^2+^, Co^2+^, Ni^2+^, and Cu^1+^ in the stable *P_Fe_*^0^, *P_Co_*^0^, *P_Ni_*^0^, and *P_Cu_*^−1^, respectively.

The *d*-band center is determined by the orbital alignment and occupation of the TM-3*d*. To account for the effects of *n*–*p* codoping on the *d*-band centers, we further calculated the spin-resolved states of density (PDOSs) of the BiOCl systems before and after *n*–*p* codoping to illustrate the orbital alignment and occupation. Based on the crystal field theory, the degeneracy of the TM-3*d* orbital states is broken in a process dependent on the molecular symmetry. As shown in Figure 1b,c, the TM ion is located at the center of a square plane formed by four nearest neighboring oxygen atoms. In this local symmetry, the TM_3*d* orbitals split into four groups *d_xz_* + *d_yz_*, *d_xy_*, *d_z_*^2^, and *d_x_*^2^*_−y_*^2^. In the neutral *Bi_TM_*^0^, the oxidation states of the TM dopant are all +3. As shown in Figure 6a, the *d* electron configuration obeys Hund’s rule in *Bi_Fe_*^0^. Five spin-up *d* orbitals are fully occupied, while five spin-down *d* orbitals are entirely empty. In the *Bi_Co_*^0^, *Bi_Ni_*^0^, and *Bi_Cu_*^0^, the *d* electron configurations break Hund’s rule as shown in Figure 6a. In detail, four spin-up *d* orbitals and only the spin-down *d_xz_* + *d_yz_* orbital are occupied in the Co_3*d*, when the electron configuration of Co is changed from 3*d*^7^4*s*^2^ to 3*d*^6^4*s*^0^ after losing three valence electrons in the *Bi_Co_*^0^. In the *Bi_Ni_*^0^, four spin-up *d* orbitals and three spin-down *d* orbitals (*d_xz_* + *d_yz_* orbitals and *d_xy_* orbital) are occupied to form the 3*d*^7^4*s*^0^ configuration. In the *Bi_Cu_*^0^, the Cu_3*d* orbitals are spin-degenerated, and spin-degenerated *d_xz_* + *d_yz_*, *d_xy_* and *d_z_*^2^ orbitals are occupied to change the 3*d*^10^4*s*^1^ to a 3*d*^8^4*s*^0^ configuration.

In the dopant–defect pairs *P_TM_*^0^, negatively charged TM dopants and positively charged *V_Cl_*^+1^ are stabilized by the electrostatic interaction of the *n*–*p* codoping. The electron accumulation changes the oxidation state of the TM dopant from +3 to +2. Compared with the electron configuration in the *Bi_Tm_*^0^, electron accumulation is located around the TM while charge depletion forms around the *V_Cl_*^+1^ and durable electrostatic attraction is established based on the Coulomb’s law as shown in Figure 4. Accumulated electrons occupy the localized empty TM_3*d* orbital as shown in Figure 6b. In detail, spin-down *d_z_*^2^ is further occupied in the *P_Fe_*^0^ to form the 3*d*^6^4*s*^0^ configuration. In the *P_Co_*^0^, the electron from the *V_Cl_*^+1^ further occupies the spin-down *d_xy_* to form the 3*d*^7^4*s*^0^ configuration. Spin-down Ni_*d_z_*^2^ is further occupied, leading to spin-degenerated Ni_3*d* states in the *P_Ni_*^0^, while the spin degeneration of the Cu_3*d* states is broken along with the spin-up d_x_^2^_−y_^2^ being occupied. Above all, newly occupied TM_3*d* orbitals are localized and remain at the Fermi level in the *P_TM_*^0^, gathering both the spin-up and spin-down TM_3*d* orbitals together and bringing them to the Fermi level. As a result, the *d*-band centers are shifted to the Fermi level compared with those in the Bi*_TM_*^0^. For the *P_Cu_*^−1^ as shown in Figure 6b, the added electrons also accumulate around the Cu SACs and fill into Cu_d_x_^2^_−y_^2^, inducing spin-degenerated Cu_3*d* orbital states and localized half-occupied d_x_^2^_−y_^2^. A stronger localized characteristic of Cu_3*d* is formed around the Fermi level, further shifting the *d*-band center to the Fermi level.

The electron accumulation around the TM SACs plays an important role in changing the *d*-orbital alignment and shifting the *d*-band center in the *P_TM_*. In contrast to the *Bi_Fe_*^−1^, the reducing capacity in the *Bi_TM_*^−1^ is weaker than that in the *P_TM_*. To account for the reduced capacity in the *Bi_TM_*^−1^, we investigate its *d* orbital alignment and electron configuration in detail. The electron accumulation is also formed around the *Bi_Fe_*^−1^ as shown in Figure S7a. The spin–polarized PDOS of TM_3*d* in the *Bi_TM_*^−1^ is shown in Figure S8a. The *Bi_Fe_*^−1^ keeps in line with the *P_Fe_*^0^ on the *d*-orbital alignment, the 3*d*^6^4*s*^0^ configuration, and even Hund’s rule, resulting in a similar *d*-band center and ΔG for the CO_2_ absorption. Although electron accumulation occurs around the *Bi_Co_*^−1^ as shown in Figure S7b, the 3*d*^7^4*s*^0^ configuration stays the same as that in the *P_Co_*^0^, but the spin–polarized *d*-orbital alignments exhibit a dramatic change compared with those in the *P_Co_*^0^. Five spin-up orbitals are occupied in the *Bi_Co_*^−1^ as shown in Figure S8a, while only four spin-up orbitals are occupied in the *P_Co_*^0^ (the same as in the *Bi_Co_*^0^) as shown in Figure 6. Such variation changes the high spin state (3*μ*_B_) to a low spin state (1*μ*_B_) as well as induces greater broadening of Co_3*d* orbital states and then a more negative *d*-band center in the *Bi_Co_*^−1^. The electron localization is weakest in the *Bi_Ni_*^−1^ as shown in Figure S7c. As a result, although the electron configuration in the Ni_3*d* is same as the cases in the *P_Co_*^0^, the energy level and broadening of Ni_3*d*, which also influence the *d*-orbital alignment, are extended to a low energy level in the *Bi_Ni_*^−1^, resulting in a much lower *d*-band center than that in the *P_Co_*^0^. The *Bi_Cu_*^−1^ possesses the same orbital alignment, orbital occupation, and *d*-band center as the *P_Cu_*^0^ as shown in Figure S8d. Therefore, it possess a similar ΔG for CO_2_ absorption. The stronger reducing capacity of the *P_Cu_*^−1^ derives from the accumulation of additional electrons around the Cu SACs. On the other hand, compared with the *Bi_TM_*^0^, *Bi_Fe_*^−1^, *Bi_Ni_*^−1^, and *Bi_Cu_*^−1^ upshift the *d*-band center because additional electrons accumulate around the TM SACs and occupy more 3*d* orbitals. In contrast to the *Bi_Co_*^0^, the *Bi_Co_*^−1^ moves the *d*-band center downward with negative energy following the transition of spin states. Consequently, the electron accumulation plays a key role in the *d*-orbital alignment and the *d*-band center. The transition of spin states also influences the *d*-band center.

### 3.4. Discussion

**Figure 7:**
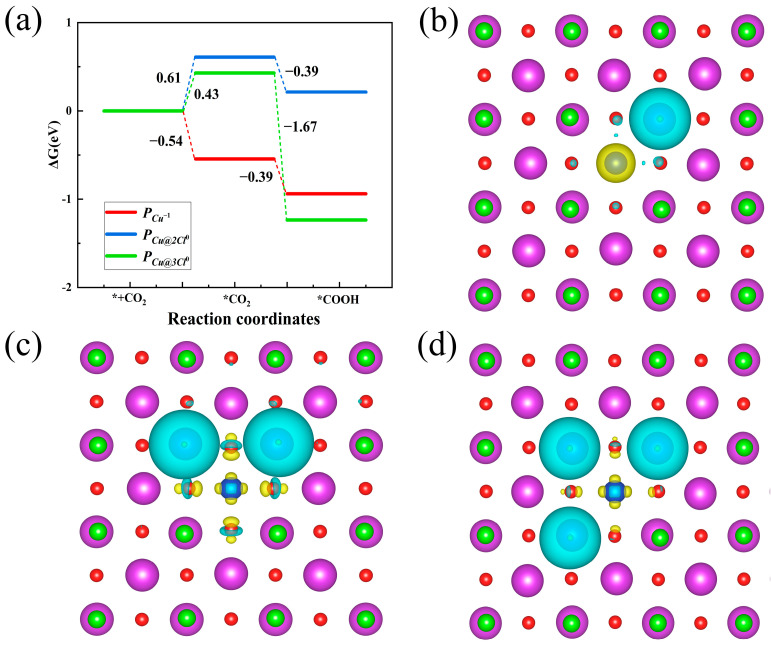
(**a**) Gibbs free energy profiles of the *P_Cu_*^−1^, *P_Cu@2Cl_*^0^, and *P_Cu@3Cl_*^0^. (**b**–**d**) depict the charge density redistribution of the *P_Cu_*^−1^, *P_Cu@2Cl_*^0^, and *P_Cu@3Cl_*^0^, respectively. The isovalue is set to 0.03 *e*/Å.

Finally, the effect of *Cl* multivacancies on the *n*–*p* codoping is discussed. Taking the most sable *P_Cu_*^−1^ as an example, the *P_Cu@2Cl_* and *P_Cu@3Cl_* are pairs with double and ternary *Cl* vacancies, as shown in Figure S1. Both the *P_Cu@2Cl_* and *P_Cu@3Cl_* exhibit lower formation energy than the *P_Cu_*^−1^. However, a high Δ*H_f_* hinders the stability and solubility of *V_2Cl_* and *V_3Cl_*, as shown in Figure S2. The solubility of both the *P_Cu@2Cl_* and *P_Cu@3Cl_* is lower than that of the *P_Cu_*^−1^. In the *e*-rich condition, both the *P_Cu@2Cl_* and *P_Cu@3Cl_* exhibit the charge state of *q* = 0, as shown in Figure S2b (denoted as *P_Cu@2Cl_*^0^ and *P_Cu@3Cl_*^0^). The ΔG profiles in Figure 7a exhibit obvious high free energy (0.61 eV and 0.43 eV) for CO_2_ absorption at both the *P_Cu@2Cl_*^0^ and *P_Cu@3Cl_*^0^ sites, which is higher than that at the *P_Cu_*^−1^ and even higher than that at the *V_Cl_*^+1^. Relatively high *d*-band centers lead to a weak reducing capacity as shown in Table 1. Obviously positive charge accumulation around the *P_Cu@2Cl_*^0^ and *P_Cu@3Cl_*^0^ is also observed as shown in Figure 7c,d, shielding the electron transfer to absorbed CO_2_ and decreasing the reducing capacity. Consequently, the *P_Cu_*^−1^ enhances the CO_2_ absorption, and doping the TM on the unlocked Bi region is not an efficient strategy to facilitate the CO_2_RR.

## 4. Conclusions

In summary, *n*–*p* codoping engineering is introduced to account for the modulation of photocatalytic CO_2_ reduction on a BiOCl-based cathode by using first-principles calculation. *n*–*p* codoping is established via the Coulomb interactions between positively charged TM SACs and the negatively charged *Cl* vacancy (*V_Cl_*) in the dopant–defect pairs. Based on the formation energy of charged defects, we find neutral dopant–defect pairs for the Fe, Co, and Ni SACs (*P_TM_*^0^) and the q = −1 charge state of the Cu SAC-based pair (*P_Cu_*^−1^). The electrostatic attraction of the *n*–*p* codoping in pairs strengthens the stability and solubility of TM SACs and stabilizes the electron accumulation around the TM SACs. Accumulated electrons occupy the localized TM_*d* orbital and change the *d*-orbital alignment, gathering the *d*-orbital states and shifting the *d*-band center toward the Fermi level and enhancing the reducing capacity of TM SACs based on the d-band theory. As a result, the CO_2_ absorption is improved with the enhancement of charge transfer and the decrease in Gibbs free energies. Besides the electrostatic attraction of the *n*–*p* codoping, additional electrons in the *P_Cu_*^−1^ also accumulate, surrounding Cu SACs and forming a half-occupied *d_x_*^2^*-_y_*^2^ state, which further upshifts the *d*-band center and improves photocatalytic CO_2_ reduction. For the pairs consisting of TM SACs and Cl multivacancies *P_TM@nCl_* (n > 1), their concentration is limited due to the metastability of *Cl* multivacancies. The positively charged center around the TM SACs blocks the charge transfer, indicating that doping the unlocked region with TM atoms does not facilitate CO_2_ reduction.

## Figures and Tables

**Table 1:** Calculated TM–C bond length (Å) for absorbed *CO_2_ (d_(*CO2)_) and *COOH (d_(*COOH)_), bond angle of absorbed *CO_2_ molecule (θ(°)), and *d*-band center (eV). “/” indicates that the distance between physically absorbed CO_2_ and the activation site is beyond 3.50 (Å).

Defect	d(*CO_2_) (Å)	d(*COOH) (Å)	d-Band Center (eV)	Θ (°)
*Bi_Fe_* ^0^	/	1.91	−2.54	179.75
*Bi_Fe_* ^−1^	1.90	1.83	−1.26	145.77
*P_Fe_* ^0^	1.90	1.83	−1.27	147.15
*Bi_Co_* ^0^	/	1.85	−1.81	179.53
*Bi_Co_* ^−1^	/	1.80	−2.45	148.32
*P_Co_* ^0^	1.97	1.81	−0.83	147.70
*Bi_Ni_* ^0^	/	1.86	−3.85	179.66
*Bi_Ni_* ^−1^	/	1.81	−2.09	179.15
*P_Ni_* ^0^	3.31	1.84	−1.46	177.90
*Bi_Cu_* ^0^	/	1.94	−2.81	179.77
*Bi_Cu_* ^−1^	/	1.93	−2.31	179.18
*P_Cu_* ^0^	/	1.93	−2.31	179.13
*P_Cu_* ^−1^	1.97	1.88	−1.45	153.71
*P_Cu@2Cl_* ^0^	/	1.88	−3.12	149.73
*P_Cu@3Cl_* ^0^	/	1.85	−1.99	179.40

## Data Availability

Data are contained within the article and Supplementary Materials.

## Supplementary Materials

The following supporting information can be downloaded at: https://www.mdpi.com/article/10.3390/nano14141183/s1, Figure S1: Schematic diagrams of atomic structure of the *P_TM@2Cl_* and *P_TM@3Cl_*; Figure S2: Formation energy of *V_nCl_*, *P_Cu@nCl_*, *P_Co@nCl_* depending on the *E_f_*; Figure S3: Δ*H_f_* of the *Bi_TM_* (rel lines) and *P*_TM_ (blue lines) at *Cl*-poor limit for (a) Fe, (b) Co, (c) Ni and (d) Cu, respectively; Figure S4: Gibbs free energy (ΔG) profiles for *V_Cl_*^+1^ and BiOCl, respectively; Figure S5: Charge transfer between surface defect sites and absorbed CO_2_ in BiOCl with (a) *Bi_F_*_e_^0^, (b) *Bi_Co_*^0^, (c) *Bi_Ni_*^0^, (d) *Bi_Cu_*^0^, respectively.; Figure S6: Charge transfer between surface defect sites and absorbed CO_2_ in BiOCl with (a) *Bi_Fe_*^−1^, (b) *Bi_Co_*^−1^, (c) *Bi_Ni_*^−1^, (d) *Bi_Cu_*^−1^, respectively; Figure S7: Polarization charge density in the (a) *Bi_Fe_*^−1^, (b) *Bi_Co_*^−1^, (c) *Bi_Ni_*^−1^, (d) *Bi_Cu_*^−1^, respectively; Figure S8: (a) pdos of the *Bi_TM_*^−1^ and (b) pdos of the *P_Cu@2Cl_*^0^, *P*_Cu@3Cl_^0^ and *P*_Cu@2Cl_^+1^. See the supplementary material for detailed results (such as formation energy, Gibbs free energy, and charge density redistribution).
