# Supplementary material for: Modulation of Photocatalytic CO2 Reduction by n–p Codoping Engineering of Single-Atom Catalysts
Source: Nanomaterials (Basel). 2024 Jul 11;14(14):1183. doi: 10.3390/nano14141183 (PMC11280387; doi:10.3390/nano14141183)
Supplement: Supplementary file 1 [file nanomaterials-14-01183-s001.zip › nanomaterials-3070790-supplementary.pdf]

SUPPLEMENTARY MATERIAL

for

Modulation of photocatalytic CO_2 reduction by *n-p* codoping engineering on single-atom catalysts

Guo-wei Yin[1], Chun-xiao Zhang[1,2,*], Yun-dan Liu [2,*], Yu-ping Sun [1,*] and Xiang Qi [2]

[1]School of Physics and Optoelectronic Engineering, Shandong University of Technology, Zibo, Shandong Province 255000, China
[2]Hunan Key Laboratory of Micro-Nano Energy Materials and Devices, Xiangtan University, Xiangtan 411105, China

**Corresponding author*

E-mail addresses: zhangchunxiao@sdut.edu.cn (C. Zhang), liuyd@xtu.edu.cn (Y. Liu), sunyuping@sdut.edu.cn (Y. Sun)

1. Figures

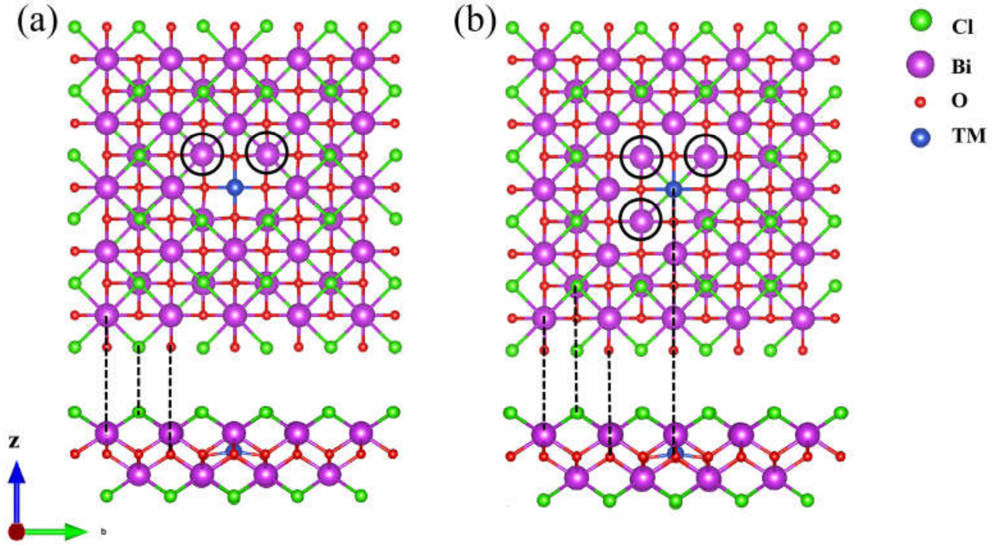

Figure S1. Schematic diagrams of atomic structure of the $P_{TM@2Cl}$ and $P_{TM@3Cl}$

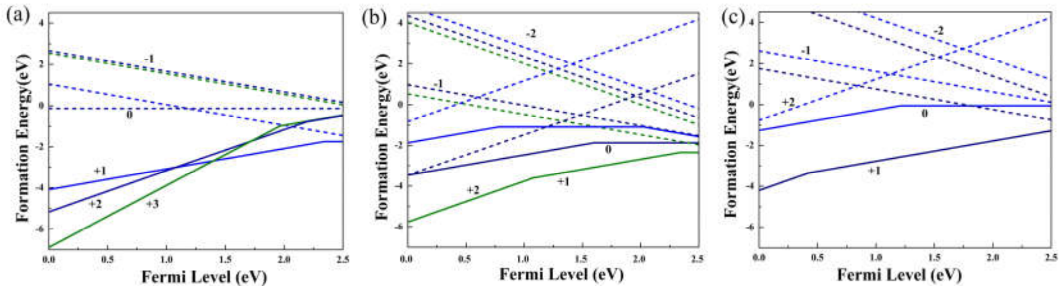

Figure S2. Formation energy of V_{nCl}, $P_{Cu@nCl}$, $P_{Co@nCl}$ depending on the E_f. The blue, navy-blue and olive-green lines refer to n =1, 2 and 3, respectively.

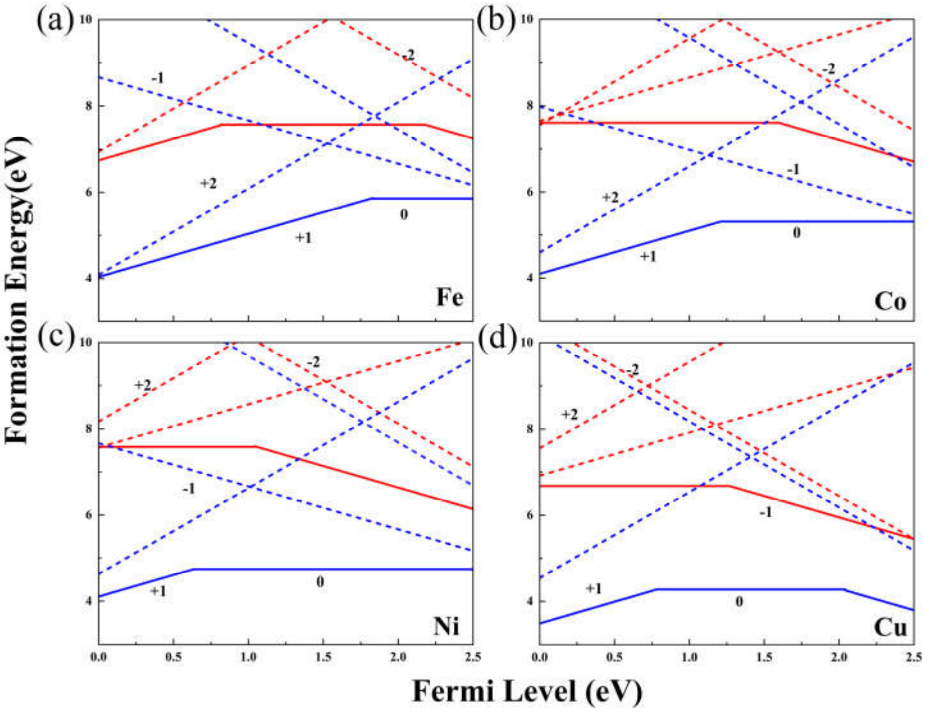

Figure S3. ΔH_f of the Bi_{TM} (rel lines) and P_{TM} (blue lines) at *Cl*-poor limit for (a) Fe, (b) Co, (c) Ni and (d) Cu, respectively. The numerical notations refer to the charge states.

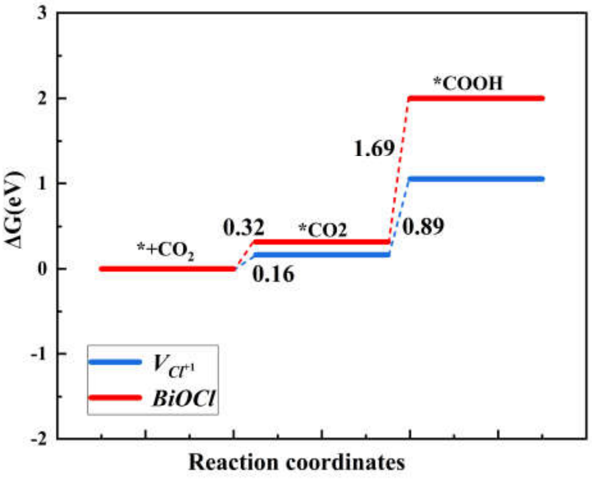

Figure S4. Gibbs free energy (ΔG) profiles for V_{Cl}^{+1} and *BiOCl*, respectively.

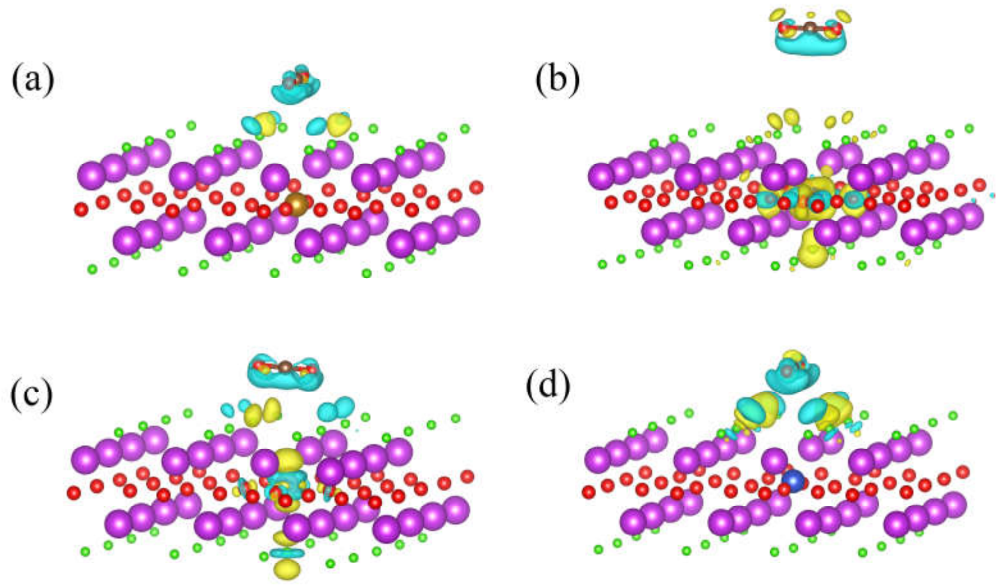

Figure S5. Charge transfer between surface defect sites and absorbed CO_2 in BiOCl with (a) Bi_{Fe}^0, (b) Bi_{Co}^0, (c) Bi_{Ni}^0, (d) Bi_{Cu}^0, respectively.The isovalue is set to 0.0003 e/Å.

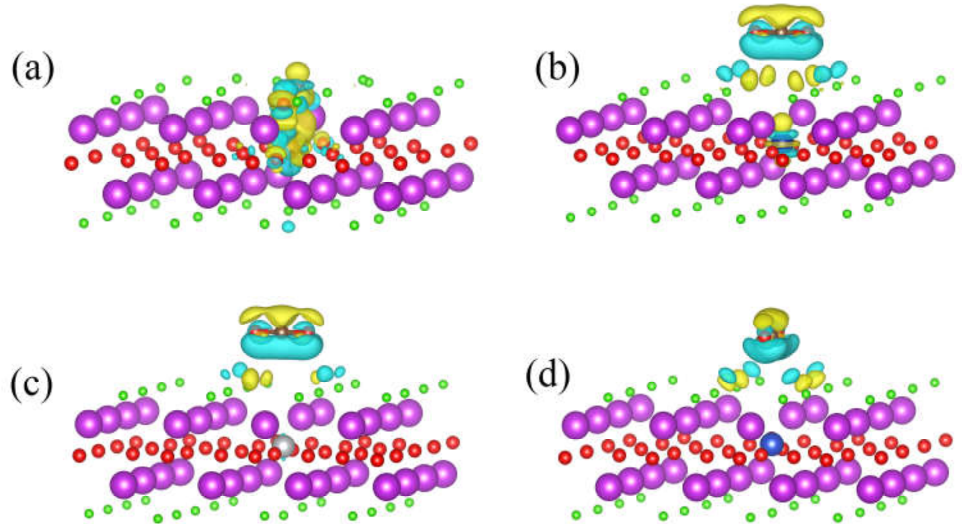

Figure S6. Charge transfer between surface defect sites and absorbed CO_2 in BiOCl with (a) Bi_{Fe}^{-1}, (b) Bi_{Co}^{-1}, (c) Bi_{Ni}^{-1}, (d) Bi_{Cu}^{-1}, respectively.. The isovalue is set to 0.0003 e/Å.

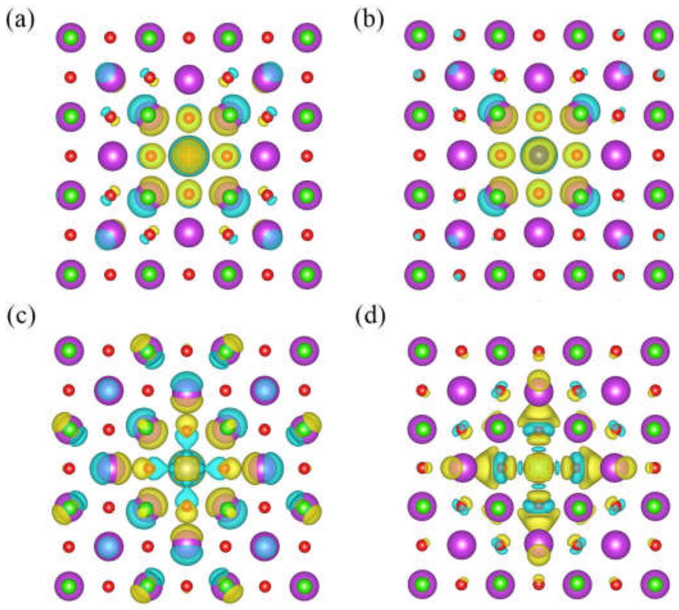

Figure S7. Polarization charge density in the (a) Bi_{Fe}^{-1}, (b) Bi_{Co}^{-1}, (c) Bi_{Ni}^{-1} and (d) Bi_{Cu}^{-1}, respectively.The isovalue is set to 0.03 e/Å.

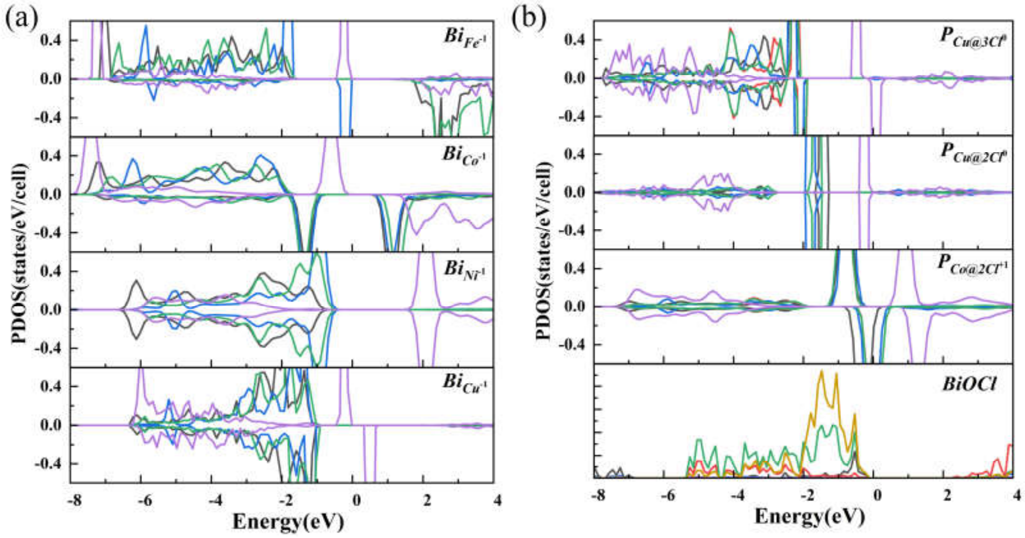

Figure S8. (a) pdos of the Bi_{TM}^{-1} and (b) pdos of the $P_{Cu@2Cl}^{0}$, $P_{Cu@3Cl}^{0}$ and $P_{\mathrm{Cu@2Cl}}^{+1}$. The orbital-resolved pdos of BiOCl is given for comparison.
